# Clinical application of boron neutron capture therapy for cancer treatment: A systematic review

**DOI:** 10.1002/pro6.70031

**Published:** 2025-11-14

**Authors:** Ling Zhou, Yuming Chen, Meng Peng, Xiumao Yin, Huanqing Liang, Jieming Mo, Wan Zhang, Zhigang Liu

**Affiliations:** ^1^ Cancer Center The Tenth Affiliated Hospital, Southern Medical University (Dongguan People's Hospital) Dongguan Guangdong China; ^2^ Dongguan Key Laboratory of Precision Diagnosis and Treatment for Tumors Boron Neutron Capture Therapy Engineering Technology Research Center of Dongguan The Tenth Affiliated Hospital, Southern Medical University Dongguan Guangdong China

**Keywords:** boron neutron capture therapy, cancer treatment, heavy‐ion therapy

## Abstract

Boron neutron capture therapy (BNCT) combines boron drug delivery and heavy‐ion therapy. The key factors in the application of BNCT are high‐quality neutron beams and boron‐containing compounds. It can be used to treat gliomas, meningiomas, melanomas, and recurrent head and neck cancers. To promote the research and development of accelerator‐based neutron sources, increase the tumor targeting capability of boron drugs, and improve BNCT efficacy in treating malignancies, this review provides an overview of the development of boron‐containing compounds and the progress made in clinical studies using BNCT.

## Graphical abstract

This review provides an overview of the development of boron‐containing drugs, and the progress made in clinical studies of BNCT, in order to promote the research and development of accelerator‐based neutron sources, increase the tumor‐targeting of boron drugs, and improve BNCT efficacy to treat malignancies.

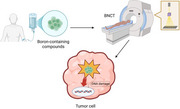

## 1 BACKGROUND

**FIGURE 1:**
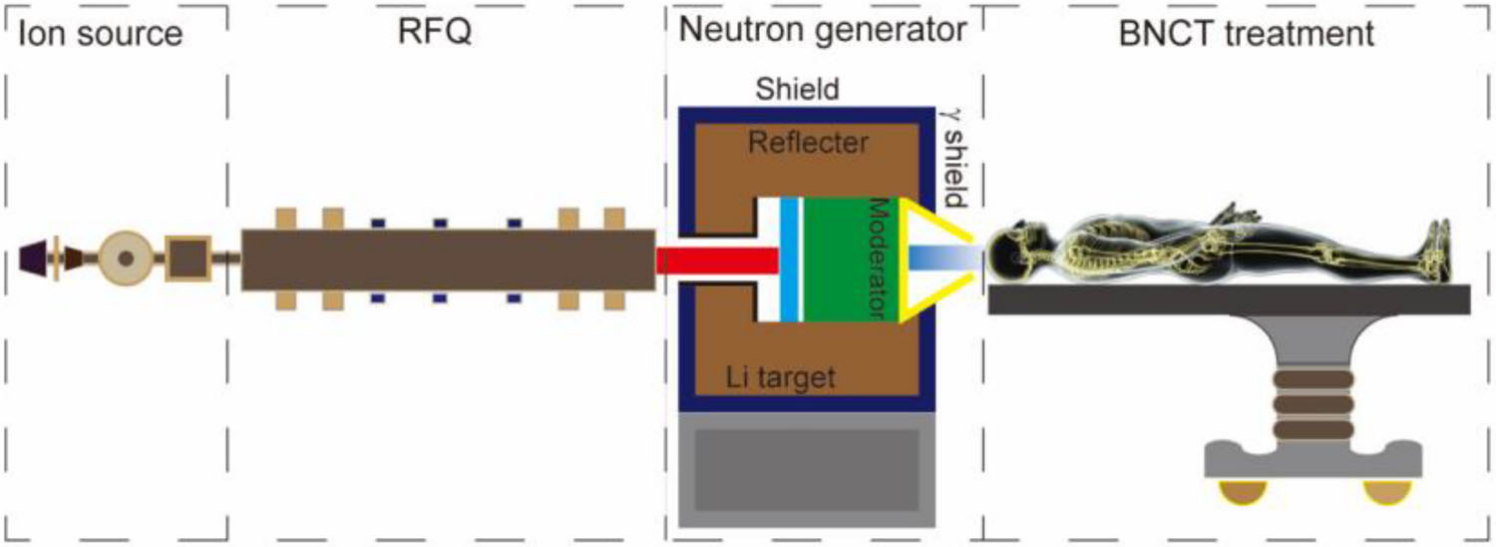
Schematic view of the BNCT system. The principle of the device is that a radio frequency quadrupole (RFQ) accelerator provides a proton beam, passing through the beam transmission line and impinging on a lithium (Li) target to produce high‐flux neutrons, the tumor can be treated under the control of the treatment software.

Boron neutron capture therapy (BNCT) is a radiation therapy that combines neutron irradiation and boron‐containing targeted drugs, has the potential to target malignant tissue at the cellular level. When neutrons collide with ^10^B, α particles and ^7^Li nuclei with short ranges of 9–10 µm and 4–5 µm are produced, respectively, which have little effect on the surrounding normal cells in tissues.^1^ Consequently, BNCT selectively eliminates cancer cells with little damage to peritumoral normal tissue compared to conventional radiation therapy.^2^ A schematic of an accelerator‐based BNCT (AB‐BNCT) system is shown in Figure 1.

## 2 THE BIOLOGICAL MECHANISM OF BNCT

BNCT causes cell death by releasing particles after ^10^B capture of thermal neutrons, and high linear energy transfer (LET) particles induce complex and irreparable deoxyribonucleic acid (DNA) double‐strand breaks (DSBs). Defects in DNA DSB repair genes can increase the sensitivity to BNCT. At 2 h following neutron irradiation, Chinese hamster ovary (CHO‐K1) cells contained 36.5%‐42.8% less tumor protein p53 binding protein 1 and gamma H2AX (γ‐H2AX) compared with that at 30 min post irradiation; however, in xrs‐5 cells foci levels decreased by 58.4%‐69.5% compared with those at 30 min post‐irradiation, and the amount of γ‐H2AX after treatment related to the effect of BNCT.^3^ Sato et al. found that dynamic changes in lymphoid‐restricted membrane protein (LRMP) levels may be related to the cellular responses to BNCT.^4^

Compared with γ‐rays, in the treatment of glioma cells, BNCT achieved a higher relative biological effectiveness, which may be related to increased Bcl‐2 expression and decreased Bax expression.^5^ BNCT treatment of melanoma cells resulted in marked changes in the extracellular matrix (ECM) and decreased cyclin D1 levels, indicating that necrosis, apoptosis, and cell cycle arrest were involved in the observed cell death.^6^, ^7^ Huang et al. found that DNA damage and repair can impact the anticancer effects of BNCT in radiotherapy‐resistant hepatocellular carcinoma cells.^8^

BNCT can also improve the immunogenicity of the tumor microenvironment. Trivillin et al. were the first to observe the distant effects of BNCT in an ectopic colon cancer model using BDIX rats. In this study, P‐boronophenylalanine (BPA) and Bacillus Calmette‐Guérin (BCG) were subcutaneously injected, and the left leg was irradiated with neutrons. The results showed that the tumor volume in the right leg decreased, and the main effector of the abscopal effect was BCG.^9^ Yoneyama et al. found that BNCT inhibited tumor growth in a bladder cancer model, with increased Annexin1 expression in tumors and infiltration by CD8‐positive lymphocytes.^10^ In addition, peripheral blood mononuclear cells (PBMCs) switched to an antitumor phenotype with decreased interleukin‐10 (IL‐10) levels and increased levels of IL‐12 after BNCT treatment.^11^

## 3 THE MEDICINAL USE OF BORON‐CONTAINING COMPOUNDS

The key to developing boron carriers lies in three main factors: low toxicity toward normal tissue and high tumor‐targeting; the tumor tissue concentration of ^10^B should range from 20–35 µg ^10^B/g; the concentration ratio of ^10^B between tumor and normal tissue (T/N) and between tumor and blood (T/B) should be more than 3–5.^12^ Soloway et al. synthesized sodium borate (BSH), which does not cross the blood‐brain barrier (BBB); therefore, its concentration in normal brain tissue is relatively low.^13^ However, ^10^B passively spreads from the blood to brain tumor sites through the damaged BBB in malignant brain tumors.^14^ BPA contains more boron than BSH and is preferentially taken up by melanoma cells through interactions with the melanin synthesis pathway.^15^ BPA can be taken up by amino acid transporters, whose expression is increased in most head and neck cancers. Consequently, BPA was approved in Japan in 2020 for the treatment of advanced and recurrent head and neck cancer.^16^

It is difficult for a single drug to act as a boron delivery agent in all types of tumors; therefore, drug combinations can improve delivery efficiency. Preloading with levodopa (L‐DOPA) resulted in a significantly enhanced BPA concentration in C6‐glioma cells, but without a significant change in normal blood and brain samples, suggesting that, for malignant gliomas suitable for treatment with BNCT, BPA accumulation can be enhanced by L‐DOPA.^17^ Increasing the dose and prolonging the infusion of BPA can improve the median survival time (MST) of patients with glioblastoma multiforme (GBM) treated with BNCT.^18^ Clinical studies have demonstrated that changing the route of administration and BBB disruption (BBB‐D) can enhance the absorption of BSH and BPA. Suzuki et al. analyzed 12 patients with head and neck cancer who underwent BNCT, and the results showed that the curative effect of intra‐arterial injections was significantly better than that of intravenous administration.^19^ Kusaka et al. demonstrated the effectiveness of cerebrospinal fluid (CSF) administration for BNCT. Compared to intravenous administration, CSF administration enhanced the concentration range of BPA in tumor tissues and increased the ^10^B concentration ratio in T/N.^20^ Common delivery methods for BPA include one‐step and two‐step infusions (OSI and TSI). A recent study showed that for OSI, the range of ^10^B concentrations in tumor tissue increased to 68.98% after continuous infusion. The use of TSI stabilized the ^10^B concentration in the blood. Thus, drug combination and delivery optimization can significantly enhance the efficacy of BNCT.^21^

Many studies have focused on the molecular design of BPA and BSH derivatives (Table 1).^22^, ^23^, ^24^, ^25^, ^26^, ^27^ Fujimoto et al. synthesized a small molecule, glucose‐BSH, which is indicated for patients with high CA19‐9 pancreatic cancer receiving BNCT treatment with a combination of glucose and BSH.^28^ Fujimura et al. produced a polyarginine peptide (polyR)‐conjugated BSH and reported that proteins of the translational machinery and CD44 were the main intracellular and extracellular targets of BSH‐polyR, respectively. BNCT‐mediated cell death induced by BSH‐polyR occurs specifically in cells expressing CD44 Consequently, BSH‐polyR may further contribute to the therapeutic optimization of BNCT in glioma stem‐like cells.^29^ SCID mouse models of heterotopic U87 glioblastoma were treated with BNCT, in which the group treated with liposome‐encapsulated BSH showed markedly slower tumor growth than the BPA and BSH groups and showed a better long‐term effect than the other groups. Further modifications to liposome‐mediated boron delivery could improve treatment outcomes.^30^ Iguchi et al. constructed BSH fused to short arginine peptides (1R, 2R, or 3R). Positron emission tomography (PET) imaging showed high uptake of BSF‐3R in the tumor region, suggesting that BSH‐3R could be used as an ideal clinical boron compound for BNCT.^31^ Other derivatives of BSH, such as BSH‐encapsulating and transferrin (TF)‐conjugated polyethylene glycol liposomes, can increase the uptake of boron in tumors and can be used as new targeted drugs for BNCT to treat cancer, inhibit tumor growth and improving survival.^32^ Zaboronok et al. synthesized elemental boron nanoparticles (eBNPs), which significantly decreased the proliferative ability of cells compared to BPA.^33^

**TABLE 1:** Novel boron‐containing compounds

Boron‐containing compounds	Interpretation
**Derivatives of BSH**	
aza‐BODIPY/^10^B‐BSH	A fluorescent aza‐BODIPY/^10^B‐BSH compound
BSH‐encapsulating TF‐PEG liposomes	BSH‐encapsulating, transferrin (TF)‐conjugated polyethyleneglycol liposomes
BSH‐polyR	Poly‐arginine peptide (polyR)‐conjugated BSH
Liposome BSH	Liposomal BSH
**Derivatives of BPA**	
BADB	Boronophenylalanine‐amide alkyl dodecaborate
eBNPs	Elemental boron nanoparticles
BPA‐f	Boronophenylalanine‐fructose
**Other novel boron delivery drugs**	
Carborane‐TAT@HA	Carborane‐TAT@hyaluronic acid
MMT1242	α‐d‐mannopyranoside
2'‐F‐RNA aptamer GL44	Nucleic acid aptamers
10BSGRF NPs	A boron nanoparticle nanomedicine (10BSGRF NPs), comprising fluorescein isothiocyanate (FITC) surface‐labeled with an RGD‐K peptide

Abbreviations: BADB, Boronophenylalanine‐amide alkyl dodecaborate; BPA, p‐boronophenylalanine; BSH, sodium borate; eBNPs, Elemental boron nanoparticles; HA, hyaluronic acid; PolyR, Poly‐arginine peptide; TF‐PEG, transferrin‐conjugated polyethyleneglycol.

Tsurubuchi et al. developed boron‐containing α‐d‐mannopyranoside, MMT1242, which demonstrated a high rate of uptake, showed a wide intracellular distribution in vitro, had a longer retention in the tumor, a higher T/N ratio, and lower toxicity compared with BSH or BPA. Furthermore, MMT1242 injected 24 h before irradiation could significantly inhibit tumor growth in mice.^34^ Vorobyeva et al. reported that nucleic acid aptamers can carry ^10^B into tumor cells. The human glioblastoma U‐87 MG cell‐specific 2'‐F‐RNA aptamer GL44 was used for boron delivery, and the results showed that GL44 loaded with boron specifically reduced U‐87 MG cell viability, which proved the principle of using aptamers to specifically deliver ^10^B in BNCT.^35^ A cyclic arginine‐glycine‐aspartate (RGD)‐functionalized maleimide‐functionalized closo‐dodecaborate‐albumin conjugate has also been reported as a promising selective drug for gliomas.^36^ In vitro biodistribution studies showed that AB‐type Lactosome (AB‐Lac) particles significantly accumulated in syngeneic breast cancer tumor cells in mice within 72 h after injection, reaching a maximum uptake rate at 12 h. T/N and T/B ratios remained stable and high within 72 h, thus demonstrating that AB‐Lac particles could be used as boron drugs for BNCT.^37^ Carborane‐TAT@hyaluronic acid (HA) induced a high boron uptake rate by tumor tissue via enhanced permeability and retention effects and increased the CD44 receptor binding capacity of HA, thus meeting the requirements for BNCT.^38^ Folic acid derivatives have low cytotoxicity, high water solubility, and an excellent ability to deliver boron to tumor cells in vitro, displaying high potential as promising boron delivery agents for tumors.^39^ Kuthala et al. found that a boron nanoparticle nanomedicine (10BSGRF NPs) comprising fluorescein isothiocyanate (FITC) surface‐labeled with an RGD‐K peptide could cross the BBB to selectively target brain tumors and deliver high doses of boron, with a T/B ratio of 2.8. 10BSGRF NPs increased the contrast of magnetic resonance imaging (MRI) to aid in the diagnosis of the location, size, and progression of brain tumors and effectively inhibited brain tumors under the guidance of MRI, thereby prolonging the mouse half‐life by 17 days (22 days in the untreated group vs. 39 days in the treated group).^40^ A recent study reported that an innovative apoferritin‐based nanohybrid showed increased boron uptake by mesothelioma and breast cancer cells, suggesting its potential application in BNCT.^41^ In addition, imaging examinations play an important role in BNCT. High‐intensity focused ultrasound can enhance BPA uptake,^42^, ^43^ especially when positron‐labeled L‐(18) F‐(10) BPA is used to evaluate its distribution using PET, which provides a reference for a BNCT treatment plan.^44^ ^18^F‐labeled BBPA produces high‐contrast tumor images on PET. However, it can also be used as a theranostic agent in BNCT.^45^

## 4 THE CLINICAL APPLICATION OF BNCT

### 4.1 Gliomas

BNCT comprises targeted radiation therapy and can be used to treat infiltrating, recurrent, and metastatic tumors such as gliomas,^46^, ^47^, ^48^, ^49^, ^50^ meningiomas,^51^, ^52^, ^53^, ^54^ melanomas,^55^, ^56^, ^57^ recurrent head and neck tumors,^16^, ^58^, ^59^, ^60^, ^61^ and other cancers.^62^, ^63^ (Table 2) Gliomas are one of the most common types of primary brain tumor and have a unique anatomical structure and biological characteristics. High‐grade gliomas are highly malignant and relapse easily, resulting in poor prognosis, with an MST of 12–15 months.^64^ Glioma stem cells show resistance to chemotherapy and radiotherapy and may be the source of malignant glioma recurrence.^65^ Therefore, clinical treatment of glioma is difficult. Although comprehensive treatment of glioma has made some progress in recent years, patient survival has not significantly improved, and traditional radiotherapy inevitably results in additional complications such as radiation‐induced brain injury, suggesting that new medical techniques should be explored.

**TABLE 2:** Clinical studies of BNCT for tumors

Main Researcher	Treatment time	Type of tumors	Number	^10^B	Curative effect
Chadha^49^	1994‐1995	Malignant glioma	10	BPA	The mOS was 13.5 months.
Kawabata^48^	2002‐2007		21	BSH, BPA	The OS was 20.7 months and the MST was 15.6 months.
Miyatake^46^	2002‐NA		22	BSH, BPA	The mOS was 10.8 months after BNCT.
Kawabata^50^	2016‐2018		27	SPM‐011	The mOS was 18.9 months.
Henriksson^47^	2001‐2003		30	BPA	The mOS was 14.2 months.
Haapaniemi^60^	2006‐2012	Head and neck tumor	9	BPA	The mOS was 13.3 months after BNCT
Wang^61^	2010‐2013		17	BPA	67% of patients receiving D80 > 40Gy achieved CR.
Hirose^59^	2016‐2018		R‐SCC (8)R/LA‐nSCC (13)	BPA	The 2‐year OS rates were 58% and 100% in R‐SCC and R/LA‐nSCC.
Kankaanranta^16^	2003‐2008		30	BPA	The 2‐year OS and PFS rates were 30% and 20%.
Hirose^77^	2020‐2021		47	^18^F‐FBPA	The 1‐year and 2‐year disease‐free survival rates were 34.6% and 26.6%, respectively.
Koivunoro^58^	2003‐2012		79	BPA	25 (36%) achieved CR, 22 (32%) achieved partial response, 17 (25%) achieved stable disease, and 5 (7%) had progressed.
Miyatake^52^	2005‐NA	Meningioma	7	^18^F‐BPA‐PET and methionine‐PET	Radiographic improvements
Kawabata^53^	2005‐2011		20	BPA	The mOS values after BNCT and diagnosis were 14.1 and 45.7 months, respectively.
Takeuchi^54^	2005‐2014		33	BPA	The mOS values after diagnosis and BNCT were 67.5 and 24.6 months.
Takai^51^	2005‐2019		44	BPA	The mOS values after BNCT and diagnosis were 29.6 and 98.4 months, respectively.
Menéndez^57^	2003‐2007	Melanoma	7	BPA	Overall response rate was 69.3%.
Hiratsuka^56^	2003‐2014		8	BPA	6/8 patients achieved CR.
Fukuda^55^	1987‐2002		22	BPA	16/22 patients achieved CR.

Abbreviations: BPA, p‐boronophenylalanine; BSH, sodium borate; CR, complete response; mOS, median overall survival; mPFS, median progression‐free survival; NA, Not Available.

The Stupp protocol is the standard treatment for GBM and involves temozolomide combined with radiotherapy.^66^ BNCT is a potential treatment for gliomas because glioma stem‐like cells (GSLCs) can take up BPA.^67^ Xiang et al. synthesized TMZB, a ^10^B‐boronated derivative of temozolomide. Compared with traditional BPA, the boron concentration ratios in T/B and T/N in the TMZB group were 3.8 ± 0.2 and 2.9 ± 1.1, respectively. In a mouse model of glioblastoma, BNCT combined with TMZB resulted in 58.2% tumor shrinkage at 31 days post‐BNCT compared with 35.2% shrinkage in the traditional BPA group; thus, TMZB‐based BNCT has promising potential for the treatment of glioblastoma.^68^

Radiotherapy is one of the primary treatment methods for malignant gliomas. The long‐term survival of patients who received X‐rays showed extensive cortical atrophy, neuronal damage, and BBB dysfunction, particularly in tumor scars. However, the brain and BBB in patients after BNCT were relatively normal, as revealed by light and electron microscopy examinations, and early imaging findings showed a reduced enhancement effect and decreased peritumoral edema.^69^ Nakagawa et al. found that BNCT inhibits tumor growth by inhibiting macrophage proliferation, which may be related to the early phase regression of peritumoral edema.^70^

Kawabata et al. reported 27 patients with recurrent malignant gliomas treated with AB‐BNCT, including 24 patients with glioblastoma (GB). The primary endpoint was survival rate at 1 year, Secondary endpoints included progression‐free survival (PFS) and overall survival (OS). This study showed a 1‐year survival rate of 79.2% and median OS (mOS) of 18.9 months in patients with recurrent GB, while those in the JO22506 trial ^71^ were 34.5% and 10.5 months, respectively. The most common adverse event was brain edema, which was relieved with bevacizumab.^50^ Miyatake et al. reported that four patients with glioma were treated with bevacizumab after BNCT. Three patients with grade III disease survived after BNCT for 14, 16.5, and more than 23 months, while one patient with grade IV disease survived for more than 26 months. BNCT combined with bevacizumab improved symptoms and prolonged survival time.^72^ Sköld found that the efficacy of BNCT was better than that of traditional radiotherapy, especially in patients with non‐methylation of the *MGMT* gene by comparing the survival data of three groups of patients who received BNCT, conventional radiotherapy, or radiotherapy plus temozolomide (TMZ).^73^ Kawabata et al. evaluated the efficacy of BNCT combined with X‐ray therapy (XRT) in treating patients with GBM. Ten patients in group 1 were treated with BNCT, and 11 patients in group 2 were treated with XRT at 20–30 Gy after BNCT. The results showed that the MSTs of the patients in group 1 and 2 were 14.1 and 23.5 months, respectively.^74^ Thus, patient survival could be prolonged using BNCT + XRT compared to BNCT alone; however, the best combined treatment still requires further exploration.^75^

### 4.2 Recurrent head and neck tumors

Approximately 15–50% of patients with head and neck cancer treated with surgery and/or radiotherapy develop local recurrence.^76^ Locally recurrent head and neck tumors are usually unresectable, and patients have poor prognosis. In a phase II clinical study (JHN002), BNCT was found to be more effective than chemotherapy, radiotherapy, or immunotherapy for treating recurrent head and neck tumors.^59^ Koivunoro et al. evaluated the efficacy of BNCT in recurrent head and neck squamous cell carcinoma and included 79 inoperable patients treated with BNCT; 25 (36%) had complete remission (CR), 22 (32%) had partial remission (PR), 17 (25%) had stable disease (SD) that lasted for 4.2 months (median), and 5 (7%) experienced progression. The median post‐treatment follow‐up was 7.8 years, with a 2‐year PFS rate of 38% and an OS rate of 21%.^58^ Hirose et al. conducted a large‐sample study to retrospectively explore the efficacy of BNCT in treating recurrent head and neck cancers, and the results showed an overall response rate of 74%.^77^ Aihara et al. analyzed the efficacy in three patients with salivary gland carcinoma. All patients achieved CR within 6 months of treatment, with a median duration of 24 months, and mOS time of 32 months. Two patients died of distant metastasis, but no serious adverse reactions of grade III or more occurred among all patients.^78^ Kankaanranta et al. conducted a single‐center phase I/Il clinical trial in which BNCT after conventional photon therapy was used to treat 30 patients with locally recurrent and inoperable head and neck tumors. Among the 29 patients who were evaluated, 22 showed a response to BNCT, among whom 6 patients had stable tumors and 1 patient progressed. The median PFS (mPFS) time of all patients was 7.5 months.^16^ Haapaniemi et al. reported on nine patients with recurrent laryngeal cancer who were treated with BNCT. Among them, 2 patients had CR, 4 patients had PR, 2 patients had SD, and 1 patient died before the curative effect could be evaluated. The most frequently reported adverse reactions are oral pain, fatigue, and stomatitis. Thus, BNCT may benefit patients with recurrent laryngeal cancer.^60^ Haginomori et al. first used BNCT to treat recurrent squamous cell carcinoma of the temporal bones following various treatments. Tumor size was significantly reduced after 6 months of BNCT treatment.^79^ Miyaguchi et al. reported nine patients with oral cancer received BNCT, 7 achieved CR, and 2 achieved PR after treatment. The OS, disease‐specific survival, and local control rate of the patients at 1‐year were 76.2%, 76.2%, and 87.5%, respectively.^80^ Sato et al. evaluated 36 patients with hypopharyngeal/laryngeal cancer who underwent BNCT, and the results showed a CR rate of 72%. Overall, BNCT produces a good response in tumors while retaining normal cell functions.^81^

Wang et al. analyzed 17 patients attending Taipei Veterans General Hospital with recurrent head and neck tumors who received BNCT twice with an interval of 28 days. The results showed that 6 patients achieved CR and 6 patients achieved PR, the 2‐year OS and locoregional control rates were 47% and 28%, respectively, in the group receiving D80 > 40 Gy (‐Eq. Appropriate doses of BNCT are safe and effective for treating locally recurrent head and neck tumors; however, some patients experience local recurrence after treatment.^61^ Therefore, we conducted a trial of BNCT plus intensity modulated radiotherapy (IMRT) for the treatment of head and neck cancer. Seven patients were treated with IMRT at 28 days post‐BNCT, and 3 achieved CR; however, failure was still caused mainly by local recurrence.^82^ In the treatment of head and neck tumors, the performance of BNCT is similar to that of chemotherapy/targeted therapy and recurrent radiotherapy, although the treatment time is short. Thus, in the future, high‐quality clinical trials with large sample sizes should be conducted to explore BNCT combined with other treatments in patients with recurrent head and neck tumors.

### 4.3 Meningiomas

High‐grade meningiomas (grades II and III) have high recurrence and low survival rates. Currently, there are only a few studies on the treatment of meningiomas using BNCT. Tamura et al. published the first case of recurrent meningioma treated with BNCT in 2006. The patient underwent many operations and tumor progression could not be controlled. She could only move using a wheelchair before BNCT treatment, whereas after 1 week of treatment, she could walk. Twenty‐two weeks later, the patient received a second BNCT treatment because of tumor recurrence in the contralateral side, after which the tumor volume decreased from 65.6 cm^3^ to 31.8 cm^3^, indicating that BNCT could be used as one of the treatments for high‐grade meningiomas.^83^ In a retrospective analysis of 44 patients with high‐grade meningiomas treated with BNCT, the mOS values at diagnosis and post‐BNCT were 29.6 and 98.4 months, respectively. The mOS values of patients with grade II meningiomas (20 cases) and grade III meningiomas (24 cases) post‐BNCT were 44.4 and 21.55 months, respectively, and the mPFS values were 24.3 and 9.4 months, respectively. However, 22.2% of patients with high‐grade meningiomas experienced local recurrence.^51^ Kawabata et al. analyzed 20 patients with recurrent meningiomas who underwent BNCT after surgery and radiotherapy. The MST after BNCT and initial diagnosis were 14.1 and 45.7 months, respectively. Additionally, facial pain and hemiplegia significantly improved after BNCT.^53^ Miyatake et al. found that most patients with meningiomas had a high boron uptake rate, and their clinical symptoms and imaging findings significantly improved after BNCT treatment; however, some patients experienced pseudo‐progression. Transient tumor enlargement was observed in 27.3% (3/11) of the patients with malignant meningioma within 3 months of BNCT, which was thought to be related to tumor necrosis in the subacute phase after BNCT.^84^ Radiation therapy is effective for unresectable meningiomas, and BNCT is effective and safe as an alternative salvage treatment for patients with recurrent meningiomas who received radiotherapy in the past.^85^ In the future, BNCT combined with other treatments such as photodynamic therapy (PDT), could be considered a novel therapy for malignant meningiomas.^86^

### 4.4 Melanomas

Skin melanomas can also be treated with BNCT. BNCT rarely causes morphological changes in normal melanocytes, whereas melanoma cells treated with BNCT display marked changes in their ECM and a significant decrease in cyclin D1 levels.^6^ Nude rats were implanted with the human melanoma cell line MRA 27 intracerebrally, followed by intraperitoneal injection of BPA into the nude rats at 30 days post‐implantation, after which the rats were irradiated with neutrons. The MST of the untreated rats was 44 days, while the MST of rats in the 2.73 Gy and 3.64 Gy dose groups was 76 and 93 days, respectively. Among the rats treated with the highest radiation dose (10.1 Gy), 40% survived for > 300 days. No residual tumor was found in the brain histopathology of long‐term surviving rats at 8 or 16 months post‐BNCT. The authors reported the accumulation of melanin macrophages and minimal gliosis.^87^ Morita et al. confirmed that intratumoral injection of the tyrosinase gene improved the effect of BNCT on melanoma cells, suggesting that BNCT has the potential to treat melanoma.^88^ Fukuda et al. analyzed 22 cases of malignant melanoma treated with BNCT from 1987 to 2002, showing that 16 cases exhibited tolerable skin damage and 73% (16/22) of the patients achieved CR.^55^ Hiratsuka et al. treated 8 patients with cutaneous melanoma using BNCT, among whom 6 achieved CR, 2 achieved PR, and only 1 relapsed 6 years later^56^. A phase I/IL clinical study in Argentina included 7 patients with melanoma, and the total remission and stability rates were 69.3% and 30.7%, respectively, with acceptable toxicity.^57^

### 4.5 Other types of tumors

With the development of BNCT, many clinical studies have suggested its application in other types of cancers, such as lung cancer,^89^ malignant pleural mesothelioma (MPM),^62^ breast cancer,^90^ liver cancer,^63^ colorectal cancer,^91^ osteosarcoma,^92^ and lymphoma.^93^ Suzuki et al. implanted murine squamous cell carcinoma cells into the thoracic cavity to create a tumor model and showed that the survival time of mice in the BNCT group was longer than that of mice in the control group; however, the incidence of mild pulmonary fibrosis was significantly higher in the BNCT group.^94^ Researchers have demonstrated the superiority of AB‐BNCT over reactor‐based BNCT (RB‐BNCT) in the treat deep tumors.^62^ Therefore, BNCT may be a potential treatment option for inoperable MPM. A patient with metastatic lung cancer underwent BNCT twice, in the left lower and upper lobes of the lung. No radiation pneumonia or other adverse reactions were observed during follow‐up period.^89^ The main advantage of BNCT is that it is a biologically targeted therapy, which is particularly important for patients with locally recurrent breast cancer after chest wall radiotherapy. Gadan et al. proposed that BNCT could be used to treat patients with HER2‐positive locally recurring breast cancer, using immunoliposomes as boron‐carrier nanovehicles to target HER2 receptors. BPA is especially promising for the treatment of breast cancer because it is absorbed by the L‐type amino acid transporter 1 (LAT1), a protein that is highly expressed in breast cancer cells.^90^ Deng et al. developed a 10B‐containing polymer that self‐assembled with PD‐L1 siRNA to form 10B/siPD‐L1 nanoparticles for combinational BNCT‐immunotherapy and found that BNCT using 10B/siPD‐L1 nanoparticles precisely killed tumor cells in a 4T1 breast cancer tumor‐bearing mouse model while sparing adjacent T cells and inducing an effective antitumor immune response, inhibiting distant and metastatic tumors.^95^ Therefore, BNCT may improve the efficacy of tumor control by mobilizing the immune system in patients who do not achieve pathological CR after neoadjuvant chemotherapy, especially those with TNBC.^96^ In 2007, a patient with multiple hepatocellular carcinomas (HCCs) was treated with BNCT for the first time. The tumor was stable within 1 month after BNCT treatment but progressed at 3.5 months after BNCT. The patient died of liver failure resulting from tumor progression at 10 months post‐treatment.^63^ BNCT's mechanism of action in the treatment of advanced liver cancer is related to the induction of DNA damage, disruption of DNA damage repair, and increased radiation‐induced apoptosis of hepatoma cells.^8^ BNCT extended the survival of mice with pelvic tumors and did not cause serious side effects in a murine model of colorectal cancer (CRC) pelvic recurrence.^91^ A study in Argentina showed that BNCT could be used to treat rats with colon cancer and induce distant effect.^97^ Futamura et al. treated a patient with osteosarcoma of the left occipital skull, who was unable to walk or undergo surgery before treatment. However, she was able to walk again 3 weeks after the BNCT. BNCT is effective and safe for treating patients with radiation‐induced osteosarcoma who are unsuitable for surgery or other treatments.^92^ A Japanese scholar explored the possibility of using BNCT to treat primary central nervous system lymphoma (PCNSL) and found that, in viv and in vitro, lymphoma cells had a high boron uptake capacity, and the BNCT group demonstrated a stronger killing ability and prolonged survival than the control group.^93^

Clinical studies are underway worldwide (Table 3). A phase I clinical trial of Cancer Intelligence Care Systems‐1 (CICS‐1) accelerator‐based BNCT using BPA (Stella Pharma SPM‐011) for malignant melanoma and angiosarcoma was completed at the National Cancer Center Hospital in Japan (NCT04293289). The team reported that 10 patients with scalp angiosarcoma and forefinger malignant melanoma received AB‐BNCT at a dose of 18 Gy, and that the best overall response rate within 180 days was 70%.^98^ Based on the results of the phase I clinical study, the team is conducting a phase II clinical study of patients with unresectable angiosarcoma who underwent BNCT (NCT05601232). The Third Xiangya Hospital of Central South University initiated a phase I/II clinical trial of IHNI‐based BNCT for the treatment of malignant melanoma to determine the value of the IHNI in BNCT performance (NCT02759536). The estimated enrollment for the clinical trial was 30 patients, and the first patient received treatment on August 19, 2014. The patient was in CR after 24 months of follow‐up, and no obvious side effects were observed, suggesting that BNCT is an effective therapy for malignant melanoma.^99^ Taipei Veterans General Hospital uses BNCT combined with IMRT to treat patients with locally recurrent head and neck tumors after radiotherapy. The primary endpoints were the response rate and toxicities (NCT02004795). Gachon University Gil Medical Center in South Korea is conducting a clinical trial to investigate the efficacy, safety, and pharmacokinetic characteristics of BNCT in patients with recurrent high‐grade glioma. (NCT05737212). The Xiamen Humanity Hospital in China is conducting a clinical trial on the safety and efficacy of BNCT for the treatment of advanced refractory head and neck tumors or primary brain malignant tumors (ChiCTR2200066473).

**TABLE 3:** Clinical trials being carried out around the world

Organization	Type of tumors	Registration number	Country
Taipei Veterans General Hospital	Locally recurrent head and neck tumors	NCT02004795	China
The Third Xiangya Hospital of Central South University	Malignant melanoma	NCT02759536	
Xiamen Humanity Hospital	Advanced refractory head and neck tumors or primary brain malignant tumors	ChiCTR2200066473	
National Cancer Center Hospital	Malignant melanoma and angiosarcoma	NCT04293289	Japan
National Cancer Center Hospital	Unresectable Angiosarcoma	NCT05601232	
Gachon University Gil Medical Center	Recurrent high‐grade gliomas	NCT05737212	South Korea

## 5 CONCLUSIONS

In conclusion, BNCT is a promising cancer treatment method that uses heavy‐ion radiation beams to kill cancer cells with little damage to peritumoral normal tissue. It causes cell death by releasing particles after ^10^B capture of thermal neutrons, and high‐let particles induce complex and irreparable DNA DSBs. Current clinical data and research results show that BNCT is safe and effective in patients with recurrent and metastatic tumors, especially gliomas, meningiomas, melanoma, and head and neck tumors; however, its efficacy needs to be further investigated. In the future, it will be necessary not only to improve the performance of boron drugs, but also to develop new boron drug delivery methods for higher concentrations of boron at cancer sites. BNCT shows stronger immunogenicity than traditional radiotherapy, indicating its great potential for combined application in immunotherapy.^100^ The purpose of combination therapy is not only to selectively enhance the effect of radiation in cancer cells but also to minimize the adverse effects on normal cells.

## CONFLICT OF INTEREST DECLARATION

There are no conflicts of interest to declare.
